# Efficient Brain Tumor Segmentation for MRI Images Using YOLO-BT

**DOI:** 10.3390/s25123645

**Published:** 2025-06-11

**Authors:** Mengying Xiong, Aiping Wu, Yue Yang, Qingqing Fu

**Affiliations:** 1 School of Electronic Information and Electrical Engineering, Yangtze University, Jingzhou 434023, China; 2023720699@yangtzeu.edu.cn; 2 School of Computing Science and Artificial Intelligence, Suzhou City University, Suzhou 215104, China; apwu@szcu.edu.cn; 3 Huizhou Customs Port Clinic, Huizhou International Travel Health Care Center, Huizhou Customs Comprehensive Technical Center, Huizhou 516006, China; mingtian78@163.com

**Keywords:** brain tumor, computer vision, deep learning, image processing, YOLOv11, YOLO-BT

## Abstract

Aiming at the problems of inaccurate segmentation and low detection efficiency caused by irregular tumor shape and large size differences in brain MRI images, this study proposes a brain tumor segmentation algorithm, YOLO-BT, based on YOLOv11. YOLO-BT uses UNetV2 as the backbone network to enhance the feature extraction ability of key regions through the attention mechanism. The BiFPN structure is introduced into the neck network to replace the traditional feature splicing method, realize the two-way fusion of cross-scale features, improve detection accuracy, and reduce the amount of calculations required. The D-LKA mechanism is introduced into the C3k2 structure, and the large convolution kernel is used to process complex image information to enhance the model’s ability to characterize different scales and irregular tumors. In this study, multiple sets of experiments were performed on the Figshare Brain Tumor dataset to test the performance of YOLO-BT. The data results show that YOLO-BT improves Precision by 2.7%, Recall, mAP50 by 0.9%, and mAP50-95 by 0.3% in the candidate box-based evaluation compared to YOLOv11. In mask-based evaluations, Precision improved by 2.5%, Recall by 2.8%, mAP50 by 1.1%, and mAP50-95 by 0.5%. At the same time, the mIOU increased by 6.1%, and the Dice coefficient increased by 3.6%. It can be seen that the YOLO-BT algorithm is suitable for brain tumor detection and segmentation.

## 1. Introduction

Currently, the global incidence and mortality rates of tumors continue to rise. According to data from the Surveillance, Epidemiology, and End Results program between 2017 and 2021, the overall incidence rate of brain tumors and other central nervous system (CNS) tumors in the United States was 6.2 cases per 100,000 people per year [1]. The GLOBOCAN 2022 report shows that in 2022, there were 321,476 new cases of brain tumors worldwide, accounting for 1.6% of all cancer cases, ranking 19^th^ in incidence; the number of deaths reached 248,305, representing 2.6% of all cancer-related deaths, placing brain tumors 12^th^ in mortality [2].

The presence and development of brain tumors can threaten human health, cause compression and damage to human brain tissue, and interfere with normal brain function [3]. It may lead to serious consequences such as blindness, paralysis, epilepsy, and sudden death and may also cause a variety of neurological dysfunctions depending on the location and nature of the tumor, seriously affecting the quality of life of patients [4]. Therefore, early and accurate detection of brain tumors is crucial for devising effective treatment strategies [5]. At the moment, brain tumor detection primarily relies on methods such as cranial CT scans [6], brain MRI [7], brain PET scans [8], biopsies, and neurological examinations.

In the clinical environment, MRI is the main way of examining brain tumors, providing the highest diagnostic accuracy that can be achieved at present. Medical imaging technology has provided significant advantages in tumor detection [9], but there are still many challenges faced in manual analysis. Manual analysis is time-consuming and error-prone, and changes in doctors’ professional knowledge and fatigue often lead to inconsistent diagnostic results. This limitation is particularly evident when large-scale patient datasets need to be processed, and the efficiency and accuracy of manual diagnosis often fail to meet the clinical needs of rapid decision making. It can be seen that the use of deep learning technology for automated tumor detection [10] has become an important way to solve this problem.

Currently, many deep learning models are being applied to tumor detection and segmentation tasks [11]. Olaf Ronneberger et al. proposed UNet, an encoder–decoder architecture that became a milestone in medical image segmentation due to its symmetric structure and skip connections [12]. Yaopeng Peng et al. introduced UNetV2, which improves detail preservation by automatically adapting network configurations and optimizing the feature fusion mechanism [13]. Hasib Zunair et al. developed Sharp UNet, which integrates an edge enhancement module to significantly improve the sharpness of tumor boundaries [14]. Berkay Eren introduced TransUNet, a structure that combines convolutional layers with transformer architecture to enhance global context modeling [15]. Lijuan Yang proposed a novel network framework named MUNet, which focuses on multi-scale information fusion for more accurate brain tumor segmentation [16]. Zongwei Zhou et al. proposed UNet++, which redesigns the skip connections using nested and dense pathways to better capture multi-scale features and reduce the semantic gap between the encoder and decoder [17].

Despite the rich variety of UNet variants, several common drawbacks still hinder their application in clinical-grade brain tumor segmentation. For instance, while architectures like UNetV2 and UNet++ improve multi-scale feature fusion, they may struggle to simultaneously preserve boundary precision for very small lesions and capture global context for large, irregular tumors, especially under low-contrast or noisy MRI conditions. Even when edge enhancement modules (Sharp UNet) or attention mechanisms (TransUNet) are introduced, the purely convolutional backbone often remains insensitive to subtle texture variations, leading to incomplete or blurred segmentations. Moreover, these enhancements typically incur significant computational and memory overhead, making real-time inference on standard medical hardware challenging.

YOLO has demonstrated unique advantages in tumor detection tasks [18]. YOLO is a real-time object detection algorithm that divides the image into grids and performs efficient, end-to-end object detection through a single forward pass, predicting bounding boxes and class probabilities. The concept of YOLO was first introduced by Joseph Redmon and Andrew Farhadi in 2015 [19]. Nadim Mahmud Dipu implemented seven different neural network-based object detection frameworks and algorithms, including YOLOv3 PyTorch, YOLOv4 Darknet, Scaled YOLOv4, YOLOv4 Tiny, YOLOv5, Faster R-CNN, and Detectron2 [20]. Chanu Maibam Mangalleibi et al. found that CNN- and YOLOv3-based approaches showed greater potential for brain tumor classification [21]. Akmalbek Bobomirzaevich Abdusalomov achieved higher accuracy by fine-tuning the YOLOv7 model through transfer learning [22]. Yuan Zizhong made a series of improvements to YOLOv3 and YOLOv5 by introducing Efficient-Rep GFPN and a decoupled head structure into YOLOv5, enhancing its feature extraction and semantic information transmission capabilities [23]. Rafia Ahsan et al. combined YOLOv5 with 2D U-Net, enabling both the detection of different types of brain tumors and the precise delineation of tumor regions within the predicted bounding boxes [24]. Naira Elazab incorporated ResNet into the YOLOv5 framework as a feature extractor, enabling accurate classification and localization of tumors in histopathological images [25]. Karacı Abdulkadir proposed a three-stage hybrid classification framework based on YOLO, DenseNet, and Bi-LSTM, capable of classifying gliomas, meningiomas, and pituitary tumors [26]. As of 30 September 2024, the latest version, YOLOv11, has emerged, achieving significant improvements in both accuracy and efficiency.

Despite the advancements embodied in YOLOv11 as a state-of-the-art iteration of the YOLO family, its architectural optimization is primarily tailored for natural image domains. When directly applied to the task of brain tumor segmentation in medical imaging, the model exhibits several inherent limitations.

Specifically, the backbone of YOLOv11 emphasizes global semantic representation, rendering it insufficiently responsive to fine-grained boundary cues and small-scale abnormalities. This leads to frequent omission or incomplete detection of small tumors, especially under conditions of low texture contrast and blurred anatomical boundaries. Although YOLOv11 introduces certain cross-layer connections, its capacity for handling targets with drastic scale variation, which are typical in brain tumor imagery, remains inadequate, resulting in inconsistent performance across large and small tumor instances. Moreover, its reliance on bounding box regression for localization impairs its precision when delineating tumors with ambiguous or complex margins, often causing deviation in boundary alignment. Critically, the accurate interpretation of pathological regions in medical images necessitates the integration of contextual information, a capacity in which YOLOv11 is notably limited. Consequently, the model is vulnerable to misclassification induced by image noise and irrelevant artifacts.

In response to these challenges, this study introduces YOLO-BT, a multi-scale feature fusion framework built upon YOLOv11. We hypothesize that by deeply integrating hierarchical features with adaptive attention mechanisms, YOLO-BT can substantially enhance the segmentation fidelity of irregularly shaped brain tumors in MRI scans, while simultaneously reducing computational latency. Experimental validation on the Figshare Brain Tumor dataset substantiates this hypothesis; YOLO-BT not only surpasses YOLOv11 in both bounding box-level and mask-level performance metrics but also achieves significant improvements in mIOU (Abbreviations are listed in Table 1) and Dice coefficient, thereby affirming the proposed method’s efficacy in elevating both detection efficiency and segmentation accuracy in the medical imaging context. The main contributions of this work are summarized as follows:

- In this study, the network structure of YOLOv11 is improved, and the YOLO-BT algorithm is proposed. Based on the original network, the algorithm optimizes the feature extraction ability of the network, improves the accuracy and efficiency of brain tumor detection and segmentation, and improves the adaptability of the model to the morphology of brain tumors.
- Experiments are carried out on the Figshare Brain Tumor dataset, and the data performance is better than other comparison algorithms. The experimental data show that YOLO-BT performs well in dealing with brain tumors with complex shapes and large size differences and exhibits significantly improved segmentation accuracy and detection efficiency compared with traditional methods.

## 2. Materials and Methods

This section mainly introduces the design and implementation of the proposed YOLO-BT algorithm, concluding with the evaluation metrics used to assess the model’s performance, with a focus on the optimization of the model architecture. Derived from YOLOv11, several critical modifications characterize the proposed model: UNetV2 is adopted as the backbone network; BiFPN is employed for multi-scale feature fusion; and the D-LKA mechanism is introduced to augment contextual information extraction. Each module’s design rationale and implementation specifics are examined here, alongside their collective influence on the performance metrics of the model overall.

YOLOv11 introduces three major improvements based on YOLOv8: when C3k2 is set to True, the C2f module is replaced by C3k2; a new mechanism called C2PSA is proposed, which embeds a multi-head attention mechanism into the C2 module; and two depthwise separable convolution layers are added to the detection head in the head network [27].

**Figure 1:**
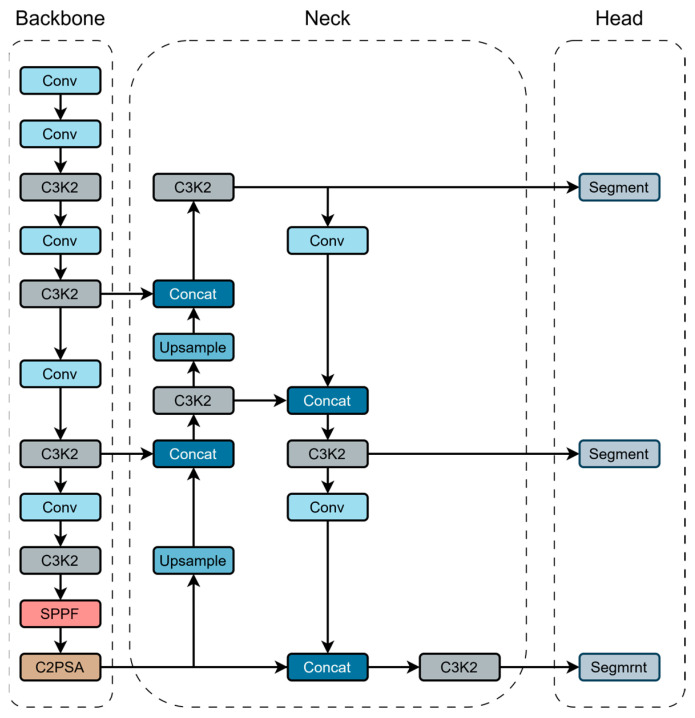
YOLOv11-Seg structure.

Considering the performance and lightweight requirements of segmentation tasks, this study selects YOLOv11n-Seg as the research baseline. The network structure of YOLOv11n-Seg is shown in Figure 1.

### 2.1. YOLO-BT Algorithm

**Figure 2:**
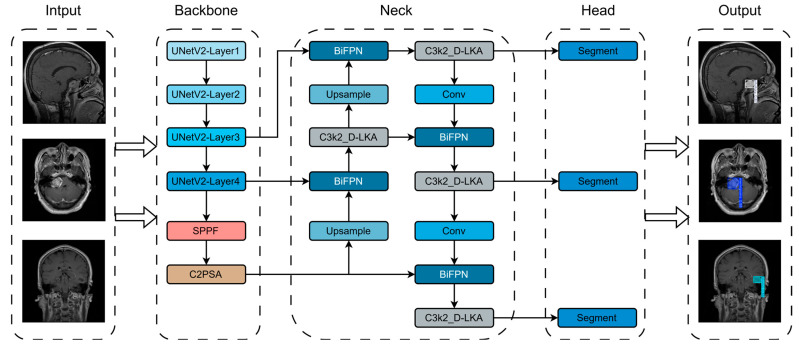
YOLO-BT algorithm architecture.

Based on YOLOv11n-Seg, we propose a more suitable algorithm for brain tumor segmentation, named YOLO-BT. The YOLO-BT architecture and its input/output are shown in Figure 2, and the network structure layers are detailed in Table 2. Compared with YOLOv11, YOLO-BT has three major architectural features:

First, the UNetV2 network is used to replace the backbone. Compared with the original fully convolutional backbone structure, UNetV2 preserves shallow spatial features through long-distance skip connections, effectively addressing YOLOv11’s insensitivity to tumor boundaries while improving segmentation accuracy.

Second, BiFPN is introduced into the Neck network. Unlike traditional Concat or FPN structures, BiFPN features bidirectional information flow and weighted feature fusion, enhancing the model’s adaptability to brain tumors of varying sizes and addressing YOLOv11’s imbalance in response to small targets.

Third, a deformable large kernel attention mechanism is integrated into the neck structure. By leveraging deformable large kernel convolution, the model’s contextual awareness of tumor regions is enhanced, leading to more accurate tumor edge detection. This significantly improves YOLO-BT’s ability to model complex-shaped and poorly defined brain tumors, thereby effectively increasing segmentation precision.

The workflow of the YOLO-BT system, along with the corresponding mathematical derivations, is presented as follows.

Let the input MRI image be denoted as $X\in R^{H\times W\times 1}$. Multi-scale feature maps are extracted using the UNetV2 encoder, defined as

(1) $$F^{\left(0\right)}=U\left(X\right)={\left\{F_{i}^{\left(0\right)}\right\}}_{i=1}^{4},F_{i}^{\left(0\right)}\in R^{H_{i}\times W_{i}\times C_{i}}$$

where the downsampling rate increases with the level i, and the number of channels $C_{i}$ follows the expansion rule $C_{i+1}=2C_{i}$.

Let the encoder output features be $\{F^{\left(k\right)}{\}}_{k=1}^{4}$. Cross-level feature interaction is constructed through BiFPN to form a pyramid structure:

(2) $$F_{\mathrm{out}}^{\left(l\right)}=B(\{F_{\mathrm{in}}^{\left(k\right)}{\}}_{k=l-1}^{l+1})\&=\sum_{k=l-1}^{l+1}\;\frac{w_{k}\times T_{k}\left(F_{\mathrm{in}}^{\left(k\right)}\right)}{\epsilon +\sum_{j}\;w_{j}}$$

where l∈{2,3,4} denotes the current level. $T_{k}(\cdot )$ is an adaptive sampling operator; bilinear upsampling is applied when k<l, and strode convolution downsampling is applied when k>l. $F_{\mathrm{in}}^{\left(k\right)}$ and $F_{\mathrm{out}}^{\left(l\right)}$ represent the input and output features at levels k and l, respectively.

Feature extraction is further enhanced by D-LKA:

(3) $$F^{dlka}={Conv}_{1\times 1}\left(Attention⊗{\left(F^{bifpn}\right)}^{\prime }\right)+F^{bifpn}$$

where $F^{bifpn}$ denotes the input feature map, $\left(F^{bifpn}\right)\prime$ denotes the intermediate feature map after convolution and GELU activation, and ⊗ represents element-wise multiplication.

The final outputs include detection and segmentation results.

Detection output:

(4) $${\hat{Y}}_{\det}=\psi_{\det}\left(F^{dlka}\right)\in R^{S\times S\times \left(5+C\right)}$$

Segmentation output:

(5) $${\hat{Y}}_{seg}=\psi_{seg}\left(F^{dlka}\right)\in R^{H\times W\times K}$$

where each S×S grid predicts 5 bounding box parameters (x,y,w,h,conf) and class probabilities for C categories. The confidence score conf∈[0,1] is output via a sigmoid activation. K denotes the number of segmentation classes.

Thus, the final formulation of the YOLO-BT system can be expressed as

(6) $$\hat{Y}=\underset{YOLO-BT}{\underbrace{\psi \left(D\left(B\left(U\left(X\right)\right)\right)\right)}}$$

where U represents the UnetV2 encoder, B denotes BiFPN-based feature fusion, D stands for D-LKA enhancement, and ψ is the multi-task prediction head.

### 2.2. UnetV2 Network

The backbone network of YOLOv11-Seg is mainly composed of convolutional layers and C3k2 modules, which are well-suited for general detection tasks. However, for brain tumor segmentation—where the environment is well defined yet highly complex—the UNetV2 network offers clear advantages. By enhancing skip connections and multi-scale feature fusion, UNetV2 enables more accurate reconstruction of segmentation details, especially when handling complex morphologies and irregular boundaries in medical images. This significantly improves segmentation precision and allows for richer contextual information capture.

**Figure 3:**
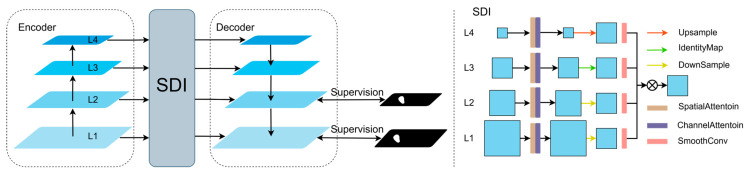
UnetV2 network architecture.

The UNetV2 architecture is shown in Figure 3 and mainly consists of an encoder, a decoder, and linked SDI modules. The encoder generates four feature maps with different resolutions, which are then processed by the SDI module, applying both spatial and channel attention mechanisms to the feature maps.

(7) $$F_{i}^{1}=\emptyset_{i}^{c}\left(\varphi_{i}^{s}\left(F_{i}^{0}\right)\right)$$

$F_{i}^{0}$ denotes the input feature map at the i level, while $\emptyset_{i}^{c}$ and $\varphi_{i}^{s}$ represent the channel attention mechanism and spatial attention mechanism at the i level, respectively. The output feature map at the i level after applying $\emptyset_{i}^{c}$ and $\varphi_{i}^{s}$ is denoted $F_{i}^{1}$.

Next, a 1 × 1 convolution is applied for downsampling to obtain the feature map $F_{i}^{2}$, where $F_{i}^{2}\in R^{H_{i}\times W_{i}\times C}$. For feature maps from different levels, adaptive average pooling, identity mapping, and bilinear interpolation are applied to generate feature map $F_{i}^{3}$ with the same resolution. These are then refined using a 3 × 3 smoothing convolution to produce the adjusted feature maps $F_{i}^{4}$.

(8) $$F_{ij}^{4}=\theta_{ij}\left(F_{ij}^{3}\right)$$

$\theta_{ij}$ represents the parameters of the smoothing convolution, and $F_{ij}^{4}$ denotes the j-smoothed feature map at the I level.

(9) $$F_{i}^{5}=H({[F}_{i1}^{4},F_{i2}^{4}{,\ldots ,F}_{iM}^{4}])$$

Finally, all feature maps at the i layer undergo element-wise Hadamard multiplication, resulting in a feature map $F_{i}^{5}$ that contains rich semantic information and details.

By integrating the attention mechanism of UNetV2 into YOLOv11 and replacing the upper portion of its backbone network, YOLO-BT’s ability to extract features from key regions is significantly enhanced.

### 2.3. Bidirectional Feature Pyramid Network

**Figure 4:**
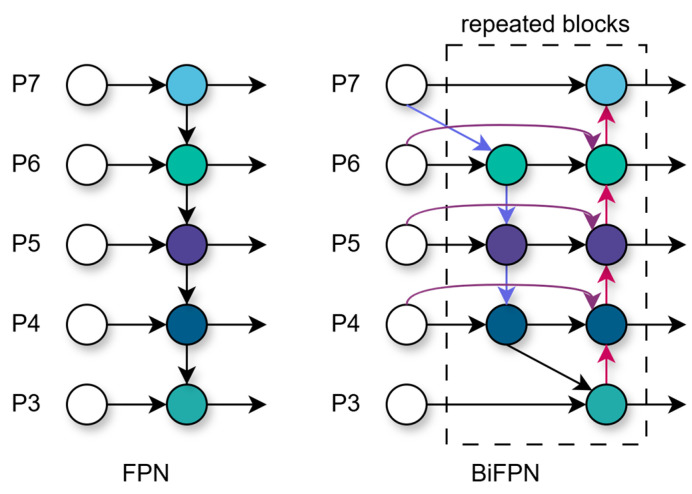
Bidirectional feature pyramid network with multi-directional connections (colored arrows) for cross-scale feature fusion.

In MRI images, most tumors vary in size, have irregular shapes, and possess blurred edges, which can lead to significant errors during detection. BiFPN addresses this issue by optimizing cross-scale connections and applying weighted feature fusion, thereby improving accuracy. It achieves comparable precision with fewer parameters and reduced computational cost, resulting in faster inference. As shown in Figure 4, BiFPN overcomes the limitations of traditional FPN’s unidirectional information flow by introducing a bottom-up path aggregation network, removing nodes with only a single input edge, and eliminating nodes with minimal contributions to the feature network, resulting in a streamlined bidirectional architecture.

BiFPN adopts fast normalized fusion to effectively merge features of different resolutions. When fusing these multi-scale features, BiFPN assigns a learnable weight to each input, enabling the network to learn the relative importance of each feature:

(10) $$O=\sum_{i}\;\frac{w_{i}}{\epsilon +\sum_{j}\;w_{j}}\cdot I_{i}$$

$I_{i}$ denotes the iii-th input feature map, and $w_{i}$ is a learnable weight. These weights are normalized into probabilities between 0 and 1, and ReLU is applied after each weight to ensure${\;w}_{i}\geq 0$. A small constant ϵ=0.0001 is introduced to prevent numerical instability. O represents the normalized output feature map:

(11) $$P_{6}^{td}=Conv\left(\frac{w_{1}\cdot P_{6}^{in}+w_{2}\cdot Resize\left(P_{7}^{in}\right)}{w_{1}+w_{2}+\epsilon }\right)$$

(12) $$P_{6}^{out}=Conv\left(\frac{w_{1}^{\prime }\cdot P_{6}^{in}+w_{2}^{\prime }\cdot P_{6}^{td}+w_{3}^{\prime }\cdot Resize\left(P_{5}^{out}\right)}{w_{1}^{\prime }+w_{2}^{\prime }+w_{3}^{\prime }+\epsilon }\right)$$

$P_{6}^{in}$ and $P_{7}^{in}$ represent the input features at levels 6 and 7, respectively. $w_{1},$ $w_{2}$, $w_{1}^{\prime }$, $w_{2}^{\prime }$, $w_{3}^{\prime }\;$ are the weight coefficients, and Resize refers to upsampling or downsampling operations. ϵ=0.0001 is a small constant to avoid numerical instability. $P_{6}^{td}$ is the intermediate feature at level 6 in the top-down path, while $P_{5}^{out\;}$ and $P_{6}^{out}$ represent the output features at levels 5 and 6 in the bottom-up path, respectively.

By integrating BiFPN into YOLOv11, replacing the original simple Concat connections, each feature level learns to adaptively fuse information from other levels through weighted fusion. This not only improves YOLO-BT’s feature representation capability but also enhances computational efficiency by pruning edge nodes and reducing the number of parameters.

### 2.4. Deformable Large Kernel Attention

**Figure 5:**
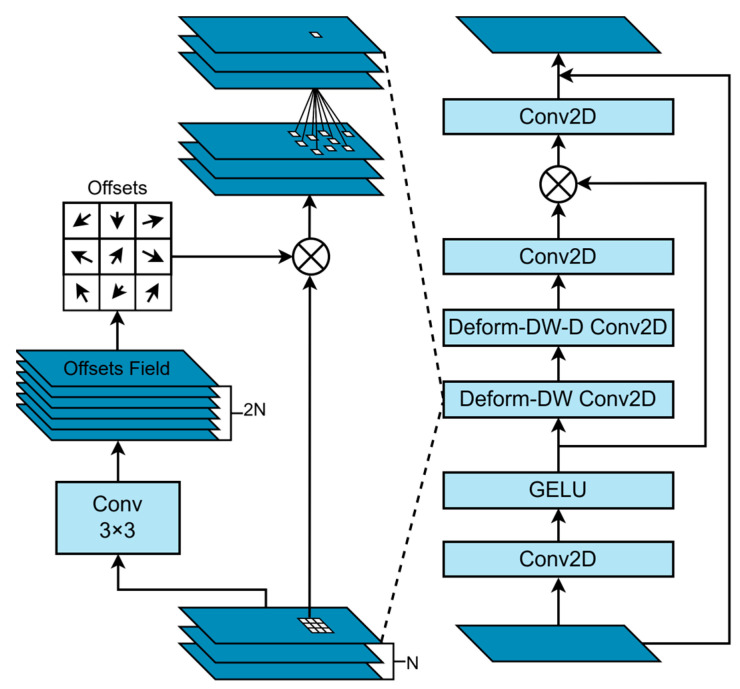
Deformable large kernel attention.

Traditional convolutional neural networks face challenges in object detection across different scales when performing image segmentation. If an object exceeds the receptive field of the corresponding network layer, it may lead to insufficient segmentation. On the other hand, a larger receptive field may incorporate background information that, when compared to the actual size of the object, can adversely influence the prediction. To address this, the D-LKA is introduced. The specific structure of D-LKA is shown in Figure 5.

Large Kernel Attention uses depthwise separable convolution layers and depthwise separable dilated convolutions to construct large convolution kernels with fewer parameters. The kernel size equations for depthwise convolutions and depthwise dilated convolutions for an input of size H×W and channels C with a kernel size of K×K are given in Equations (13) and (14):

(13) $$DW=\left(2d-1\right)\times \left(2d\;–\;1\right)$$

(14) $$DW\;–\;D=\left\lceil \frac{K}{d}\right\rceil \times \left\lceil \frac{K}{d}\right\rceil$$

where *d* is the dilation rate, DW represents the dilated depthwise separable convolution, K is the size of the input feature map, and D is the total number of blocks.

Building upon large kernel attention, the D-LKA introduces deformable convolutions. Deformable convolutions adjust the sampling grid using integer offsets, enabling free deformation. The additional convolutional layers learn the deformation from the feature map, creating an offset field. Learning deformation based on the features themselves results in an adaptive convolution kernel that improves the representation of deformed objects, enhancing the definition of tumor boundaries. The D-LKA module can be expressed as

(15) $$Attention=Conv1\times 1\left(\text{DDW-D-Conv}(\text{DDW-Conv}(F^{\prime }))\right)$$

(16) $$Output=Conv1\times 1\left(Attention⊗F^{\prime }\right)+F$$

(17) $$F^{\prime }=GELU\left(Conv\left(F\right)\right)$$

*F* represents the input feature map, where $F\in R^{C\times H\times W}$. $F^{\prime }$ denotes the intermediate feature map obtained after the convolution and GELU activation. DDW-Conv refers to the dilated depthwise separable convolution, while DDW-D-Conv represents a combination of dilated depthwise separable convolution and pointwise convolution. The symbol ⊗ denotes element-wise multiplication, and the final output is denoted as Output.

In this study, the C3k2-D-LKA module is used to replace the C3k2 module in the neck of YOLOv11, enhancing the ability of C3k2 to fuse important features and improving the segmentation of tumor lesion boundaries.

### 2.5. Experimental Evaluation Metrics

Common evaluation metrics used in YOLO include Box_P, Box_R, Box_mAP50, Box_mAP50-95, Mask_P, Mask_R, Mask_mAP50, and Mask_mAP50-95. Their formula definitions are as follows:

(18) $$Box_{Precision}=\frac{TP}{TP+FP}$$

(19) $$Box_{Recall}=\frac{TP}{TP+FN}$$

(20) $$Box_{mAP}=\frac{1}{N}\sum_{j}^{N}\;{AP}_{j}$$

(21) $$AP=\int_{0}^{1}\;p\left(r\right)dr$$

TP represents the number of samples correctly predicted as positive, FP represents the number of samples incorrectly predicted as positive, and FN represents the number of samples incorrectly predicted as negative. N refers to the number of classes, and AP stands for Average Precision, with its calculation formula shown in Equation (21). Box_mAP50 refers to the mean Average Precision at an IOU threshold of 50%, while Box_mAP50-95 represents the average AP calculated over IOU thresholds ranging from 50% to 95% with an interval of 0.005.

(22) $$Mask_{Precision}=\frac{TP}{TP+FP}$$

(23) $$Mask_{Recall}=\frac{TP}{TP+FN}$$

(24) $$Mask_{mAP}=\frac{1}{N}\sum_{j}^{N}\;{AP}_{j}$$

(25) $$Dice=\frac{2\times TP}{2\times TP+FP+FN}$$

(26) $$IOU=\frac{TP}{TP+FP+FN}\;,mIOU=\frac{1}{N}\sum_{i=1}^{N}{IOU}_{i}$$

TP denotes the total number of pixels correctly segmented as a tumor, FP indicates the number of pixels incorrectly segmented as a tumor, and FN refers to the number of pixels incorrectly segmented as non-tumor. Dice is a metric used in image segmentation tasks to evaluate the degree of overlap between the predicted results and the ground truth labels. Its calculation formula is shown in Equation (25). IOU represents the ratio of the intersection area to the union area between the predicted box and the ground truth box; the larger the value, the higher the matching degree. mIOU refers to the average IOU calculated by summing the IOU values of each class and then taking the mean. Their calculation formulas are shown in Equation (26).

## 3. Results

Concentrated in this section is the experimental verification of the YOLO-BT algorithm. Included alongside the description of datasets and configuration of experimental environments are analyses focusing on three distinct improvement modules; their impact on model performance is examined in detail. Through ablation experiments, each key component’s contribution is assessed; examples illustrate how detection and segmentation effects are evaluated, both qualitatively and quantitatively. The computational resource requirements of YOLO-BT were summarized, and an analysis of experimental results and heatmap visualizations for different tumor categories on the test set was conducted to demonstrate that the model did not exhibit overfitting. Not only does the experimental portion confirm YOLO-BT’s effectiveness for brain tumor detection and segmentation tasks, but it also lays the groundwork for subsequent optimization efforts.

### 3.1. Experimental Environment Configuration

The experimental hardware and software configurations are summarized below. Stochastic Gradient Descent is employed as the optimizer, with a learning rate of 0.01, a batch size of 8, a momentum of 0.937, and AMP enabled. The model is trained for 300 epochs. Additional training settings are provided in Table 3.

### 3.2. Dataset Overview and Preprocessing

#### 3.2.1. Dataset Source

The dataset used in this study is the Figshare Brain Tumor Dataset, provided by Southern Medical University in Guangzhou, China [28,29]. It comprises 3064 MRI images from 233 patients, with tumors categorized into three types: meningiomas, gliomas, and pituitary tumors. The distribution of tumor types is as follows: meningiomas (23.11%), pituitary (30.35%), and glioma tumors (46.54%). To effectively mitigate the risk of model overfitting, this study adopts the hold-out validation strategy, partitioning the dataset into training (2450 samples), validation (305 samples), and test sets (309 samples) with a ratio of 8:1:1 (As shown in Table 4). The training set is used for parameter learning; the validation set independently monitors the training process and triggers the early stopping mechanism, while the test set remains strictly isolated and is used solely for final performance evaluation. This strategy enables real-time detection of overfitting tendencies during training, thereby ensuring optimal model performance.

#### 3.2.2. Dataset Preprocessing

**Figure 6:**
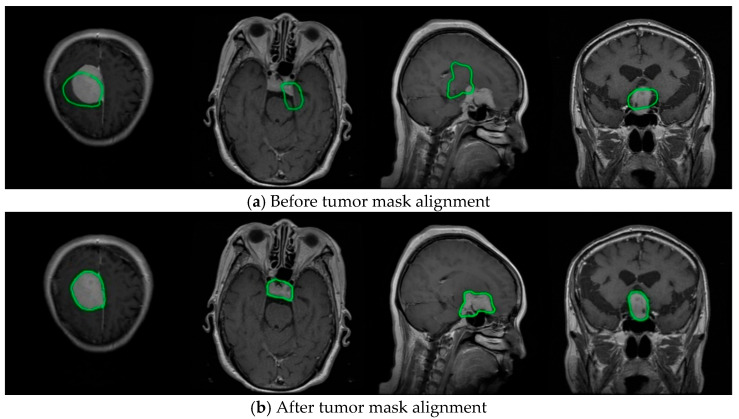
Dataset processing. (**a**) Original tumor masks (green contours) generated by YOLO before alignment; (**b**) Aligned tumor masks after registration. The green contours highlight the spatial adjustment of labels across MRI slices.

The data preprocessing procedure employed in this study addresses the misalignment between the labels and the original images in the Figshare Brain Tumor Dataset’s “.mat” files. As illustrated in Figure 6a, the .txt label files are not properly aligned with the corresponding original images. To correct this issue, an optimization approach based on OpenCV was applied. Specifically, tumor mask contours were extracted from the .mat files and normalized to ensure accurate alignment with the original images. The detailed preprocessing steps are summarized in Table 5.

The core steps of the preprocessing pipeline are as follows: loading the .mat dataset files (Step 2), iterating through each file for processing (Step 3), binarizing the tumor mask images extracted from the .mat data (Step 4), and extracting polygonal contours (Step 5). The resulting polygon coordinates are then normalized to the YOLO format (Step 6), followed by the generation of standardized label files (Step 7), thereby completing the alignment and correction of the mask annotations. Additionally, visualization and verification are performed (Step 9), in which several images are randomly selected and their processed .txt labels are overlaid on the original images to validate the accuracy of the label format.

### 3.3. Ablation Experiment Results and Analysis

To verify the segmentation performance of the YOLO-BT algorithm for brain tumors and evaluate its tumor detection ability, the ablation study was carried out using the preprocessed Figshare brain tumor dataset. Based on the YOLOv11 network, eight comparative experiments were designed, and UNetV2, D-LKA, and BiFPN modules were successively incorporated into the network for training. The network parameter configuration of YOLO-BT can be found in Section 3.1 of the paper.

According to Table 6, in the second set of experiments, after adding the UNetV2 module, the box-based Precision increased by 5.1%, while the mask-based Precision, mIOU, and Dice increased by 4.4%, 3.2%, and 1.9%, respectively, indicating that the positioning, classification, and segmentation performance of the model was improved. In the third group, after integrating the BiFPN module, the box-based and mask-based Precision both increased by 2%, showing enhanced feature extraction capabilities. In the fourth group, the performance of the model remains relatively stable after the introduction of the D-LKA module. In the fifth group, the combination of UNetV2 and BiFPN resulted in consistent improvement in all indicators. In the sixth group, the combination of UNetV2 and D-LKA resulted in a 5.3% increase in Precision, which was the highest in all groups, although the Recall rate was relatively low. In group seven, the combination of BiFPN and D-LKA did not significantly improve the performance of the model. In the eighth group of experiments, the complete YOLO-BT model was constructed by integrating the UNetV2 architecture, BiFPN, and D-LKA modules. The results demonstrate that this configuration achieves substantial improvements across all evaluation metrics, fully validating the synergistic enhancement brought by the combined modules. In candidate detection performance, YOLO-BT achieved a Precision of 92.1%, a Recall of 91.9%, mAP50 of 94.7%, and mAP50-95 of 69.2%, indicating excellent performance in both object localization and classification accuracy. Compared to the baseline model, all metrics showed varying degrees of improvement, reflecting a more balanced and stable performance. In the mask-based evaluation, YOLO-BT attained a Precision of 91.8%, a Recall of 91.5%, a mAP50 of 94.5%, and a mAP50-95 of 67.2%. Additionally, the two key segmentation metrics—mIOU and Dice coefficient—reached 86.3% and 92.6%, respectively. Compared to the baseline, this configuration improved mIoU by 6.1% and Dice by 3.6%, demonstrating stronger modeling capability for tumor boundary details and contextual semantic information. These results confirm that YOLO-BT has achieved a comprehensive breakthrough in both accuracy and robustness, representing the optimal performance among all tested models and validating the effectiveness and advancement of the proposed method for complex brain tumor detection and segmentation tasks in MRI images.

The ablation study results indicate that the proposed YOLO-BT architecture is not a mere accumulation of individual modules but rather a strategically designed model tailored to overcome the specific limitations of YOLOv11 in medical image segmentation tasks. By introducing the UNetV2 structure as the backbone, YOLO-BT significantly enhances the capability of spatial detail preservation, addressing YOLOv11’s limited sensitivity to small and irregular tumor boundaries. Furthermore, the integration of the BiFPN module across multiple hierarchical levels enables efficient bidirectional multi-scale feature fusion, which effectively mitigates the challenge of detecting tumors with large intra-class scale variations, which is a known shortcoming of YOLOv11. In addition, the incorporation of the D-LKA module strengthens the model’s global receptive field and boundary modeling capacity, allowing for better handling of blurred or complex lesion edges. Together, these three components synergistically improve the model’s representation capacity from spatial, semantic, and contextual dimensions, leading to superior performance in both detection accuracy and segmentation precision across diverse tumor types and imaging conditions.

**Figure 7:**
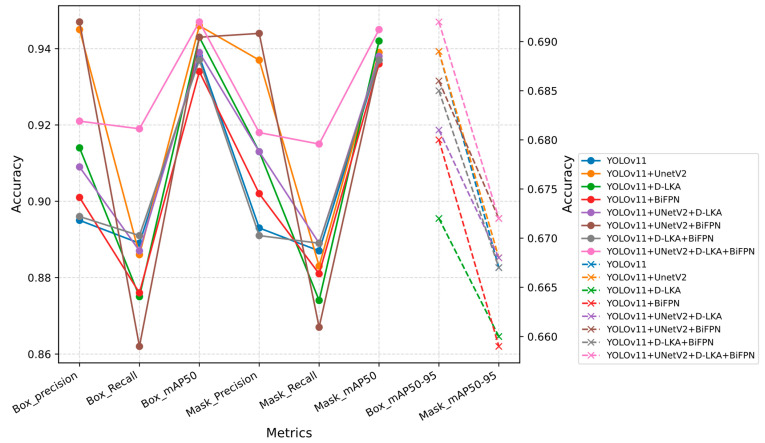
Ablation Experiment Comparison.

The comparison results of ablation experiments are shown in Figure 7. It can be seen from the figure that the YOLO-BT algorithm is more suitable for brain tumor detection and segmentation tasks. It achieves a high Recall rate while maintaining a good balance with accuracy. In addition, it produces the highest values in both Box_mAP50-95 and Mask_mAP50-95, demonstrating its superior performance.

**Figure 8:**
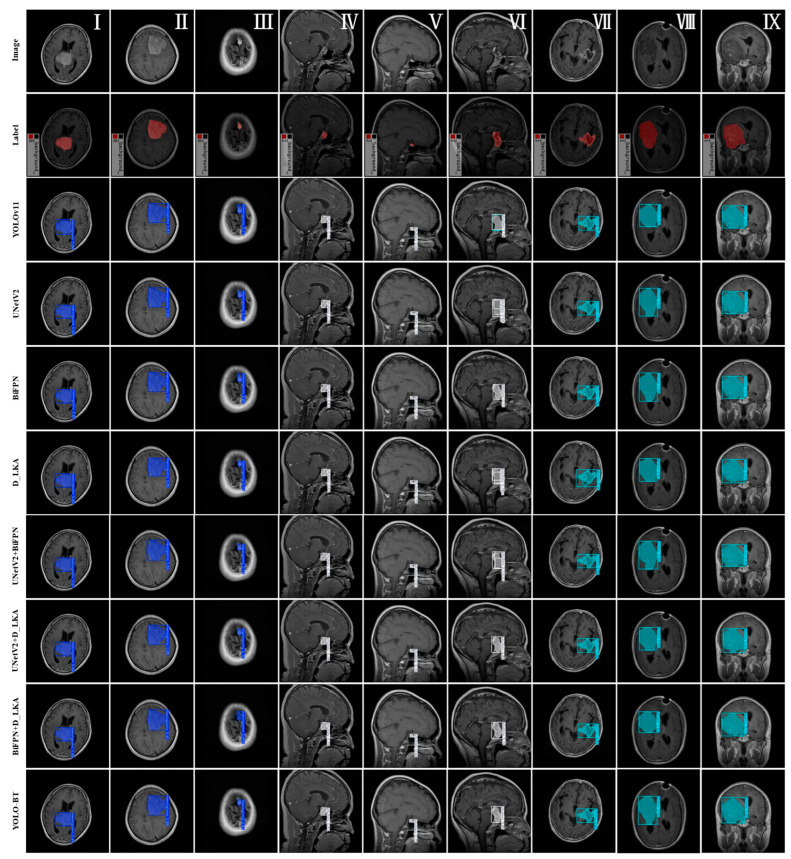
Ablation experiment test results. Figures (**I**–**III**) represent the meningioma class and are labeled as 0. Figures (**IV**–**VI**) represent the pituitary class and are labeled as 2. Figures (**VII**–**IX**) represent the glioma class and are labeled as 1.

To evaluate the impact of different modules on the brain tumor dataset, three images of each tumor type were randomly selected from the test set for testing. The results are shown in Figure 8.

After the introduction of BiFPN, the model showed more stable detection performance for tumors of different sizes. It can be seen from the results of Figure 8II,VI that the model improves the boundary detection of gliomas and meningiomas. However, the results of Figure 8IV,V are not satisfactory, indicating that compared with YOLO-BT, the detection stability of pituitary adenomas (usually small, with blurred edges) is poor. The addition of the D-LKA module leads to more accurate tumor contour segmentation and shows good performance for glioma and meningioma. However, compared with YOLO-BT, its high computational load affects the real-time detection speed. The YOLOv11 model generally shows an average detection performance, providing only rough bounding boxes. From the results of Figure 8VIII,IX the segmentation accuracy is insufficient. Although the detection confidence is relatively high, the overlap with ground truth segmentation is lower than that of YOLO-BT. In addition, the model fails to correctly classify Figure 8VI, resulting in errors. When UNetV2 is used as the skeleton, the tumor boundary becomes clearer after segmentation, as shown in the results of Figure 8V,VII,IX. However, its tumor detection ability is not as good as YOLO-BT. The UNetV2 + BiFPN model produces more stable results. From Figure 8III,IV, the model shows significant improvements in detecting small tumors compared to other models. The UNetV2 + D-LKA model provides more accurate segmentation results and performs well in glioma detection, but it requires a longer inference time and higher computing resources. The BiFPN + D-LKA model shows relatively stable test results, but in Figure 8VIII,IX, its segmentation performance for large tumors is significantly insufficient. In contrast, the YOLO-BT model shows strong performance in detecting and classifying all three tumor types (meningioma, pituitary, and glioma) with clear boundaries. In the evaluation of the nine test images, it is evident that YOLO-BT achieves the highest overlap with the ground truth segmentation results, regardless of whether the tumor is a large-area glioma or a small-region pituitary tumor. Moreover, while successfully identifying the tumor type, the model also demonstrates the highest confidence scores. It effectively combines the advantages of object detection and segmentation and achieves a good balance between accuracy and real-time performance.

### 3.4. Quantitative Evaluation

**Figure 9:**
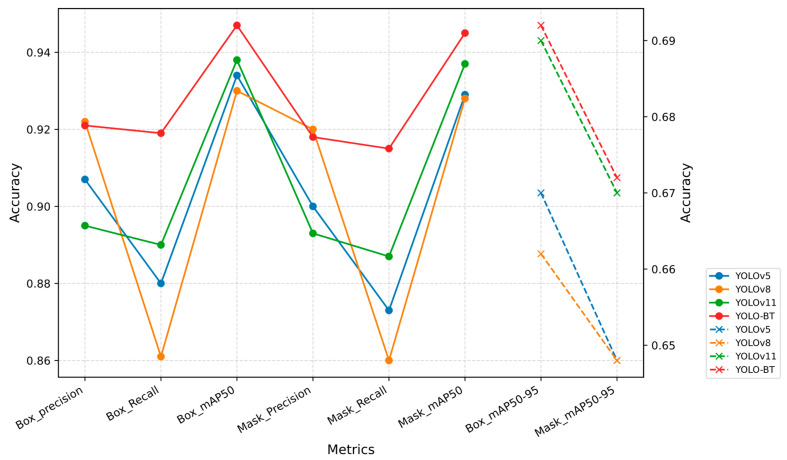
Comparison of YOLO series networks.

**Figure 10:**
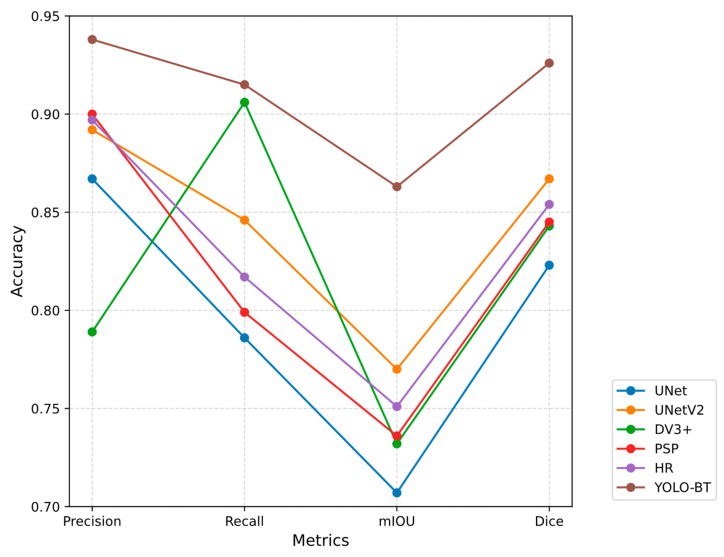
Comparison of different network models.

To validate the effectiveness of the proposed model in this study, YOLO-BT was first compared with other versions of the YOLO algorithm. The experimental results are presented in Table 7, with the corresponding performance curves shown in Figure 9. Additionally, comparisons were made with other brain tumor segmentation networks. The summarized experimental results are listed in Table 8, and visual comparison images can be found in Figure 10. The network parameter configuration of YOLO-BT can be found in Section 3.1 of the paper. Other YOLO series networks have the same configuration as YOLO-BT.

As shown in Table 6, YOLOv8 achieved the highest accuracy, indicating that the model has a low false positive rate in identifying healthy tissues. In brain tumor detection tasks, missing tumors may have serious consequences for patients, so it is important to minimize false negatives. Therefore, the main goal of the tumor detection model should be to optimize the Recall rate, even if this will slightly reduce Precision. The proposed YOLO-BT algorithm shows strong performance in both Precision and Recall. Its accuracy is only 0.1% lower than the highest value, and its Recall rate is 1.9% higher than the highest value of other models. This makes YOLO-BT the best-performing model of all test methods. The algorithm achieves a higher detection rate while maintaining accuracy close to the maximum value, which further verifies the reliability of the proposed algorithm.

Figure 9 reflects the evaluation metric scores in the comparison experiment of YOLO series networks. It can be observed that YOLO-BT outperforms other tumor detection algorithms in terms of feature extraction capability. YOLO-BT shows significant advantages in precise localization, segmentation, and controlling missed detections. The algorithm proposed in this study, YOLO-BT, demonstrates the best performance in model evaluation.

The network parameter configuration of YOLO-BT can be found in Section 3.1 of the paper. From the data in Table 8, it can be observed that UNet maintains a Precision of 86.7% and a Dice coefficient of 82.3% in its basic structure, and the overall performance is stable. However, its Recall rate and mIOU are relatively low, and it may miss the boundary segmentation of complex shape lesions and struggle with small targets or complex texture regions. The UnetV2 network showed improvement, with a Recall rate of 0.846 and an mIOU of 77.0%, indicating a higher coverage of complex lesions and a lower missed detection rate. The accuracy of UnetV2 is 89.2%, and the Dice score is 86.7%, which improves the boundary continuity while maintaining high prediction accuracy, although it may still have some local misjudgments for small lesions. DeepLabV3+ has the highest Recall rate of 90.6%, indicating that it is highly sensitive to the lesion area and effectively avoids missed detection. However, its accuracy and mIOU are relatively low, and the model may be falsely positive in complex contours or small lesion areas, resulting in a decrease in the accuracy of fuzzy boundary areas. PSPNet achieves 90.0% high accuracy with its pyramid pooling module and shows excellent performance in integrating global context information with a low false detection rate. However, its Recall rate and mIOU are relatively conservative, and it may not be able to effectively segment small lesions or blurred edges, which limits its ability to capture local details. HRNet uses multi-resolution parallel computing to achieve a good balance. The accuracy is 89.7%, the Recall rate is 81.7%, the mIOU is 75.1%, and the Dice is 85.4%. However, for highly irregular lesions, there are subtle deviations in boundary segmentation, and their adaptability to complex shapes needs to be improved. YOLO-BT is the most comprehensive and optimal model based on overall performance. By jointly optimizing object detection and segmentation, YOLO-BT significantly enhances the ability of traditional methods to process boundary details. It can accurately locate the lesion area while reducing the missed and false detection of complex shapes and small lesions, reflecting the technical advantages of global and local feature fusion. The area coverage and overlap in the segmentation task of YOLO-BT are better, and the results are closer to the real situation.

As shown in Figure 10, YOLO-BT maintains both high Recall and high Precision, demonstrating its ability to reduce false negatives while minimizing false positives, resulting in a more balanced overall performance. The mIOU and Dice scores of YOLO-BT are significantly higher than those of other models, indicating that YOLO-BT has better regional coverage and overlap in segmentation tasks, and the results are closer to the real situation.

### 3.5. Qualitative Evaluation

**Figure 11:**
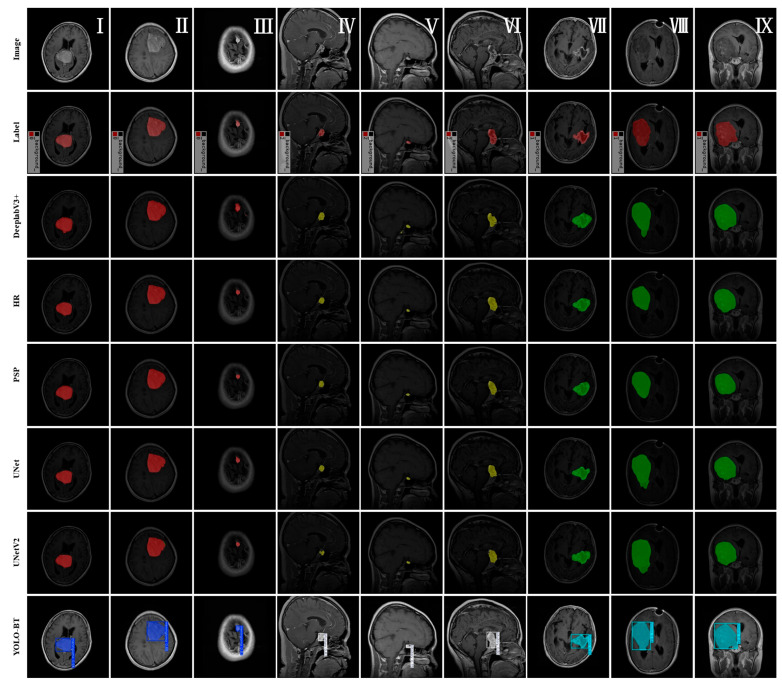
Testing results of different models. Figures (**I**–**III**) represent the meningioma class and are labeled as 0. Figures (**IV**–**VI**) represent the pituitary class and are labeled as 2. Figures (**VII**–**IX**) represent the glioma class and are labeled as 1. Apart from the YOLO-BT model, red indicates a prediction of meningioma, yellow indicates a prediction of pituitary tumor, and green indicates a prediction of glioma.

Nine random images were selected from the Figshare Brain Tumor test dataset to qualitatively evaluate the segmentation performance of the proposed algorithm and other models. Figure 11 displays the segmentation results of different methods for brain tumor regions. The figure shows, from top to bottom, the original images, the ground truth labels, and the segmentation results from the network models DeepLabV3+, HRNet, PSPNet, UNet, UNetV2, and YOLO-BT.

From the test results in Figure 11I, it can be observed that DeepLabv3+ is highly sensitive to the tumor area and can detect most of the lesion areas. For large gliomas, such as gliomas in Figure 11II, detection is effective for complete contours. However, for Figure 11VII ,IX, the segmentation results are general, and there is serious over-segmentation. For smaller lesions such as pituitary tumors, the segmentation performance is poor, and in the segmentation results of Figure 11V, there is a false detection. The segmentation results of HRNet are relatively stable, and the contour detection of meningioma and glioma is more complete. However, for more complex lesion areas, as shown in Figure 11VII, the model has a poor ability to detect tumor boundaries. Compared with YOLO-BT, it has more serious errors, and some target areas are not accurately captured. This shows that the model has moderate adaptability to complex boundaries, and the edge of the lesion is blurred. PSPNet is good at extracting global information, which can be seen from the detection results of Figure 11I,II,VII. It performs well in identifying the overall shape of meningioma and glioma, but its segmentation accuracy is low for small pituitary tumor regions. The overall segmentation performance of UNet is stable, and the boundary is clear. Compared with other models, the segmentation results of Figure 11VII are better, but there is a serious problem of false detection in Figure 11VIII. Compared with UNet, the UNetV2 model has better boundary completeness, but there are still false detections and unstable segmentation of edge regions.

YOLO-BT demonstrated outstanding performance in the detection and segmentation of all three tumor types—meningioma, pituitary tumor, and glioma. It not only accurately localizes lesion regions but also exhibits significant advantages in segmenting small targets (pituitary tumors) and irregularly shaped lesions (gliomas). Among all evaluated models, YOLO-BT consistently produced the clearest boundaries and segmentation results that most closely match the ground truth.

### 3.6. Generalization Ability Evaluation

**Figure 12:**
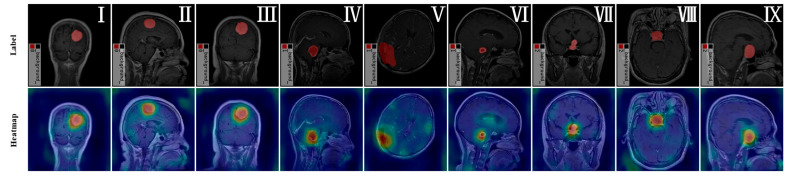
Visualization of attention heatmaps for different tumor types. Figures (**I**–**III**) represent the meningioma class. Figures (**IV**–**VI**) represent the glioma class. Figures (**VII**–**IX**) represent the pituitary class. The red-shaded regions in the top-row masks indicate manually annotated tumor areas. In the bottom-row heatmaps, the color scale ranges from cool (blue) to warm (red) activations, with red regions denoting the areas that contribute most strongly to the model’s decision for that category.

To further assess the generalization capability of the YOLO-BT model and investigate potential overfitting to the Figshare dataset, we conducted a quantitative evaluation using the held-out test set, which comprises 309 images evenly distributed across three brain tumor categories: meningioma, glioma, and pituitary tumor, as summarized in Table 9. In addition, we performed qualitative analysis by selecting nine representative samples (three per category) and visualizing the model’s attention via heatmaps, as depicted in Figure 12.

As shown in Table 9, although the test set was selected using the hold-out method and consists entirely of data previously unseen by the model, the predicted mAP50 for both bounding boxes and segmentation masks remains above 89% across all tumor categories. The overall performance is relatively balanced, indicating that the model does not suffer from overfitting to any specific category that would result in a significant performance degradation for other categories. Notably, the model demonstrates strong robustness and stable performance even for gliomas, which are characterized by high morphological variability. These results provide further evidence of the model’s strong generalization capability.

As illustrated in Figure 12, the attention heatmaps for the selected nine test images consistently show that the model concentrates its focus (indicated by red regions) on the tumor areas, while exhibiting minimal activation (blue regions) in the surrounding tissues and background. This behavior suggests that the model effectively attends to the most relevant discriminative regions for tumor detection and segmentation while suppressing irrelevant details.

Notably, no excessive activation is observed outside the tumor regions, which indicates that the model does not overfit the training data by memorizing irrelevant patterns. If the model had overfitted to the dataset, we would expect the high-response regions (in red) to be diffusely distributed across the entire image. The observed focused attention pattern thus further supports the generalization ability of the proposed model.

### 3.7. Deployment Performance Evaluation

YOLO-BT (Net 8) contains 14.36 million parameters and has a GFLOPs value of 38.6 (see Table 10 for detailed computational resource comparisons). Compared to the original YOLOv11 model (Net 1), YOLO-BT presents increased model complexity; however, it maintains high inference efficiency, with an average inference time of only 4.4 milliseconds per image on an NVIDIA RTX 4090 GPU, demonstrating its suitability for real-time segmentation tasks. Moreover, the model’s moderate size (approximately 29 MB) and reliance on standard convolutional operations facilitate compatibility and deployment across various hardware platforms, including embedded AI devices such as the NVIDIA Jetson series. Overall, YOLO-BT achieves a well-balanced trade-off between accuracy and efficiency. It delivers significant performance improvements without incurring excessive computational costs and shows strong scalability and deployment ability in real-world medical applications, particularly in resource-constrained or real-time environments.

## 4. Discussion

### 4.1. Comparative Analysis

The YOLO-BT algorithm proposed in this study demonstrates significant performance advantages in brain tumor detection tasks. Compared to the original YOLOv11, YOLO-BT incorporates a UnetV2-based backbone network that effectively fuses semantic and detailed features. This design alleviates the limitations of traditional models in extracting features from blurred tumor boundaries and significantly improves boundary localization accuracy. The BiFPN module enables dynamic, cross-level feature fusion, enhancing the model’s adaptability to tumor structures of varying scales. In addition, the D-LKA module combines the global perception ability of large convolutional kernels with the local modeling capability of deformable convolutions, significantly improving the representation of irregular tumor shapes.

While DeepLabV3+ exhibits high sensitivity to large tumor regions, it tends to suffer from over-segmentation and false positives when handling small lesions—issues that YOLO-BT addresses with greater stability. HRNet performs well in contour recognition of meningiomas and gliomas but struggles with complex and blurry boundaries, whereas YOLO-BT’s UnetV2 backbone offers a more robust solution. PSPNet can effectively identify the overall shapes of meningiomas and gliomas but lacks accuracy in segmenting small lesions and is less precise than YOLO-BT in handling tumors at multiple scales. UNet and its variant UNetV2 produce relatively clear segmentation contours and stable results, but they still face challenges such as false detections and instability in boundary predictions.

Experimental results show that YOLO-BT outperforms YOLOv11 and other mainstream detection models in key metrics such as Accuracy, Recall, mIoU, and Dice coefficient. Notably, it demonstrates superior robustness and discriminative ability in identifying small lesions, making it a more reliable and effective solution for brain tumor detection and segmentation.

### 4.2. Limitation Analysis

Despite its outstanding performance, YOLO-BT has several limitations. The proposed model has been validated and tested only on the Figshare brain tumor dataset and has not yet been evaluated on real clinical MRI scans acquired from multiple centers or imaging devices. Therefore, its practical effectiveness in clinical settings remains unverified. The model’s segmentation performance may decline in images with low resolution, motion artifacts, or noise interference. Although YOLO-BT exhibits higher computational complexity compared to YOLOv11, it still meets the requirements for deployment on medical devices. In the future, model pruning or lightweight optimization strategies may be explored to further reduce inference latency and memory consumption. Furthermore, although YOLO-BT surpasses existing methods in terms of Recall, it still carries a risk of missing very small or low-contrast lesions. Future improvements could involve incorporating Transformer-based attention mechanisms to enhance feature representation or designing more robust loss functions (e.g., focal Tversky loss) to improve sensitivity to hard-to-detect samples.

### 4.3. Future Perspectives

From a clinical application standpoint, YOLO-BT exhibits strong capabilities in precise tumor segmentation and localization, offering radiologists an efficient and accurate diagnostic aid. Integrating the model into picture archiving and communication systems could enable real-time image processing and further extend its application to multi-organ tumor analysis, including lung and breast tumors. This would support the development of intelligent diagnostic platforms across disease types and promote the real-world translation and deployment of AI technologies in healthcare.

## 5. Conclusions

To address three major challenges in brain tumor detection—namely, low sensitivity to morphological variations, inadequate multi-scale feature fusion, and inaccurate boundary localization—this study proposes the YOLO-BT algorithm, which integrates multiple machine learning techniques to achieve significant improvements, detailed as follows.

- The D-LKA module effectively mitigates the issue of blurred and irregular tumor boundaries, increasing the Dice coefficient from 89.0% to 92.6%.
- The BiFPN introduces learnable weights to dynamically fuse multi-level semantic features, resulting in notable improvements in both mAP50 and mAP50-95 on the Figshare brain tumor dataset.
- The lightweight UNetV2 backbone enhances feature representation through dual-path encoding and skip connections, improving the mIOU metric from 80.2% to 86.3%.

Experimental results demonstrate that YOLO-BT achieves a Recall exceeding 0.915, effectively reducing the likelihood of missed diagnoses and supporting clinical decision making. To fully realize the research objective of deploying a robust and generalizable brain tumor detection system in clinical settings, the following tasks are identified for future work:

- Integration of neural architecture search techniques to optimize and compress the model for deployment on resource-constrained devices;
- Validation in clinical environments, including cross-hospital and cross-device scenarios, to assess practical usability and reliability;
- Future efforts that will further advance the practical deployment of the YOLO-BT algorithm in intelligent brain tumor diagnosis.

## Figures and Tables

**Table 1:** List of abbreviations.

Name	Interpretation
YOLOv11	The 11^th^ Version of the YOLO Algorithm
C3k2	Cross-Stage Partial Kernel-optimized with K = 2
C2f	Cross-Stage Partial Two-way Feature Fusion
C2PSA	Cross-Stage Partial with Pyramid Squeeze Attention
SPPF	Spatial Pyramid Pooling Fast
D-LKA	Deformable Large Kernel Attention
BiFPN	Bidirectional Feature Pyramid Network
FPN	Feature Pyramid Network
MRI	Magnetic Resonance Imaging
CT	Computed Tomography Scan
PET	Positron Emission Computed Tomography
mIOU	Mean Intersection Over Union
Dice	Dice Similarity Coefficient
AMP	Automatic Mixed Precision

**Table 2:** YOLO-BT network structural layers.

From	n	Params	Module	Arguments
−1	1	11,940,224	pvt_v2_b1	[]
−1	1	394,240	ultralytics.nn.modules.block.SPPF	[512, 256, 5]
−1	1	249,728	ultralytics.nn.modules.block.C2PSA	[256, 256, 1]
−1	1	0	torch.nn.modules.upsampling.Upsample	[None, 2, ‘nearest’]
[−1, 3]	1	2	ultralytics.nn.modules.block.BiFPN	[2]
−1	1	49,664	ultralytics.nn.Addmodules.DLKA.C3k2_DLKA	[256, 128, False]
−1	1	0	torch.nn.modules.upsampling.Upsample	[None, 2, ‘nearest’]
[−1, 2]	1	2	ultralytics.nn.modules.block.BiFPN	[2]
−1	1	12,544	ultralytics.nn.Addmodules.DLKA.C3k2_DLKA	[128, 64, False]
−1	1	73,984	ultralvtics.nn.modules.conv.Conv	[64, 128, 3, 2]
[−1, 9]	1	2	ultralytics.nn.modules.block.BiFPN	[2]
−1	1	33,280	ultralytics.nn.Addmodules.DLKA.C3k2_DLKA	[128, 128, False]
−1	1	295,424	ultralvtics.nn.modules.conv.Conv	[128, 256, 3, 2]
[−1,6]	1	2	ultralytics.nn.modules.block.BiFPN	[2]
−1	1	312,704	ultralytics.nn.Addmodules.DLKA.C3k2_DLKA	[256, 256, True]
[12, 15, 18]	1	10,046,655	ultralytics.nn.modules.head.Segment	[3, 32, 64, [64, 128, 256]]

**Table 3:** Experimental configuration parameters.

Name	Environment Parameters
Operating System	Ubuntu 22.04
Development Framework	PyTorch 2.2.0-Ubuntu 22.04
CPU	AMD EPYC 7402 24-Core Processor (AMD, Santa Clara, CA, USA)
GPU	NVIDIA GeForce RTX 4090 24GB (NVIDIA, Santa Clara, CA, USA)
Python	3.12

**Table 4:** Dataset description.

Dataset	Train	Validate	Test	Classes
Figshare	2450	305	309	3

**Table 5:** Algorithm for image label correction.

Algorithm Process Description	
Input	Dataset file in .mat format
Output	Aligned YOLO-format Coordinates in .txt
Step 1	Begin
Step 2	Initialize the dataset files in .mat format
Step 3	Process each file individually
Step 4	Extract the tumor mask (cjdata.tumor mask) from the .mat file
Step 5	Binarize the mask image and extract polygonal contours
Step 6	Convert the contours into YOLO-format normalized coordinates
Step 7	Generate YOLO-format label files (.txt)
Step 8	End for loop
Step 9	Visualize and validate the results
Step 10	End

**Table 6:** Ablation experiment data statistics.

Net	UNetV2	BiFPN	D-LKA	Box (P	R	mAP50	mAP50-95)	Mask (P	R	mAP50	mAP50-95	mIOU	Dice)
**1**				0.894	0.890	0.938	0.689	0.893	0.887	0.934	0.667	0.802	0.890
**2**	✓			0.945	0.886	0.946	0.689	0.937	0.883	0.937	0.668	0.834	0.909
**3**		✓		0.914	0.875	0.943	0.672	0.913	0.874	0.942	0.66	0.807	0.893
**4**			✓	0.901	0.876	0.934	0.680	0.902	0.881	0.936	0.659	0.804	0.891
**5**	✓	✓		0.909	0.887	0.939	0.681	0.913	0.889	0.938	0.668	0.820	0.901
**6**	✓		✓	**0.947**	0.862	0.943	0.686	**0.944**	0.867	0.937	0.672	0.825	0.904
**7**		✓	✓	0.896	0.891	0.937	0.685	0.891	0.889	0.937	0.667	0.802	0.890
**8**	✓	✓	✓	0.921	**0.919**	**0.947**	**0.692**	0.918	**0.915**	**0.945**	**0.672**	**0.863**	**0.926**

**Table 7:** Comparative experiments of YOLO series networks.

Net	Box (P	R	mAP50	50-95)	Mask (P	R	mAP50	50-95)
**YOLOv5**	0.907	0.880	0.934	0.670	0.900	0.873	0.929	0.648
**YOLOv8**	**0.922**	0.861	0.930	0.662	**0.920**	0.860	0.928	0.648
**YOLOv11**	0.894	0.890	0.938	0.690	0.893	0.887	0.937	0.670
**YOLO-BT**	0.921	**0.919**	**0.947**	**0.692**	0.918	**0.915**	**0.945**	**0.672**

**Table 8:** Comparative experiments of different network models.

Net	Precision	Recall	mIOU	Dice
**UNet** [12]	0.867	0.786	0.707	0.823
**UNetV2** [13]	0.892	0.846	0.770	0.867
**DeeplabV3+** [30]	0.789	0.906	0.732	0.843
**PSP** [31]	0.900	0.799	0.736	0.845
**HR** [32]	0.897	0.817	0.751	0.854
**YOLO-BT**	**0.938**	**0.915**	**0.863**	**0.926**

**Table 9:** Experimental data for different tumor categories.

Category	Box (P	R	mAP50	50-95)	Mask (P	R	mAP50	50-95)
**M** **eningioma**	0.979	0.986	0.993	0.839	0.979	0.986	0.993	0.837
**G** **lioma**	0.872	0.845	0.894	0.575	0.872	0.845	0.894	0.542
**P** **ituitary**	0.913	0.925	0.953	0.661	0.903	0.914	0.948	0.637
**A** **ll**	0.921	0.919	0.947	0.692	0.918	0.915	0.945	0.672

**Table 10:** Computational complexity and inference performance.

Net	UNetV2	BiFPN	D-LKA	FLOPs (B)	Parameters	Inference Time (ms)	Memory Usage (G)	GPU Memory (MB)
1				10.4	2,835,153	1.7	1.27	855
2	P			37.8	14,072,521	4.4	1.30	967
3		P		10.8	2,923,149	2.2	1.33	861
4			P	9.8	2,701,793	2.7	1.29	859
5	P	P		37.8	14,175,121	3.5	1.24	967
6	P		P	**38.7**	14,260,441	4.0	1.38	**969**
7		P	P	14.2	3,403,117	1.9	1.19	869
8	P	P	P	38.6	**14,363,041**	**4.4**	**1.40**	967

## Data Availability

The original data presented in the study are openly available in Kaggle at https://www.kaggle.com/datasets/ashkhagan/figshare-brain-tumor-dataset (accessed on 14 November 2024).
